# Activated Desulfurized Rubber Powder/SBS/SEBS Composite-Modified Asphalt: Performance and Synergistic Modification Mechanism

**DOI:** 10.3390/polym17233113

**Published:** 2025-11-24

**Authors:** Qidong Su, Songqiao Yang, Wenjing Liu, Mingming Zhang, Aoxue Li, Dongwei Cao

**Affiliations:** 1 Research Institute of Highway Ministry of Transport, Beijing 100088, China; 2 Shanxi Road & Bridge Construction Group Co., Ltd., Taiyuan 030031, China; 3 School of Civil Engineering, Chongqing Jiaotong University, Chongqing 400074, China; 4 School of Materials Science and Engineering, Chang’an University, Xi’an 710064, China; 5 College of Traffic and Transportation, Chongqing Jiaotong University, Chongqing 400074, China; 6 CTVIC, Research Institute of Highway Ministry of Transport, Beijing 100088, China

**Keywords:** rubber-modified asphalt, modified asphalt, SBS, SEBS, rheological properties, sustainability, mechanism analysis

## Abstract

Due to the poor comprehensive performance of traditional rubber powder-modified asphalt (RA) and issues like easy segregation in rubber powder/Styrene–Butadiene–Styrene (SBS) composite-modified asphalt, the application of RA in high-grade highways is limited. This study combined SEBS (Styrene–Ethylene–Butylene–Styrene) and SBS to form SEBS/SBS (SE-S) and investigated the effect of the SE-S system on asphalt performance. The activated desulfurized rubber powder (ARP) was prepared via mechanical thermal oxidation and used to produce ARP/SE-S composite-modified asphalt (ASSA) combined with SE-S. The performance and modification mechanism of ASSA were evaluated through conventional, rheological, and microstructural tests. The results showed that SEBS improved storage stability more effectively than SBS. With SEBS:SBS = 0.4:0.6 and a SE-S content of 2–4%, the modified asphalt exhibited better overall performance. The synergy of ARP and SE-S enhances both low-temperature crack resistance and high-temperature deformation resistance, endowing ASSA with excellent viscoelastic rheological properties. The modification mechanism of ASSA was primarily physical and the changes in chemical bonds were mainly caused by decrosslinking of the rubber powder during ARP preparation. SE-S and ARP fully swelled and crosslinked in the asphalt, exhibiting excellent compatibility and endowing the ASSA with superior stability and performance.

## 1. Introduction

The escalating severity of traffic loads and environmental conditions demands asphalt binders with superior performance to resist prevalent distresses like rutting, cracking, and fatigue damage, which critically undermine pavement service life. This seriously reduces driving comfort and safety, and significantly shortens the service life of the road surface [1,2]. Improving the service performance of asphalt pavement throughout its entire life cycle has become a key research direction in the field of road engineering [3,4,5].

Meanwhile, with the development of the highway and automobile manufacturing industries, the production of waste tires continues to increase [6,7]. The global challenge of waste tire disposal, with over 1.5 billion units generated yearly and a low recycling rate, necessitates innovative upcycling solutions [8,9]. The accumulation of waste tires occupies a large amount of land and generates solid waste that is difficult to degrade. Its chemical composition and flammable properties lead to air, soil, and water pollution [10]. Moreover, the high cost and technical difficulty of recycling make waste tires an urgent “black pollution” problem that needs to be solved globally.

While utilizing crumb rubber from waste tires in asphalt is an established green technique, conventional RA suffers from limitations like poor storage stability and inadequate high-temperature performance. [11]. It can effectively reduce the accumulation of solid waste while improving the aging resistance and fatigue resistance of matrix asphalt [12,13]. However, due to the poor solubility of ordinary rubber powder in asphalt, there are disadvantages such as high viscosity, severe segregation, and insufficient high-temperature performance in modified asphalt [14,15,16]. Therefore, to enhance the reactivity and compatibility of rubber powder, researchers have employed various desulfurization, degradation, and surface activation treatments. These methods include mechanical, ultrasonic, chemical grafting, and microbial approaches [17]. Zhang et al. used mechanical methods to desulfurize rubber powder. After desulfurization, the surface active groups of the rubber powder increased, significantly improving its compatibility with asphalt [18]. Liu et al. used microwave and chemical grafting activation to treat waste rubber powder. The results showed that the storage stability and low-temperature crack resistance of activated rubber powder-modified asphalt were significantly improved [19].

Notably, while desulfurized rubber powder-modified asphalt shows significantly improved viscosity and compatibility over ordinary RA, its overall performance remains inadequate for high-grade pavements. To address this, researchers often combine rubber powder with other modifiers [20].

SBS (Styrene–Butadiene–Styrene) is a common asphalt modifier known for endowing asphalt with excellent high- and low-temperature performance. Islam et al. identified the SBS molecular structure as crucial to its modification effectiveness, noting that appropriate selection can significantly enhance both high-temperature deformation and low-temperature cracking resistance [21]. Pandey et al. reported that SBS asphalt outperforms thermoplastic polymer (TP)-modified asphalt in both rutting and cracking resistance [22]. Moreover, Song et al. found that a composite of desulfurized rubber powder and SBS offers better rutting and cracking resistance than rubber powder alone, while also reducing hydrogen sulfide emissions by approximately 45%, highlighting its environmental benefit [23]. In addition, Guo et al. demonstrated that SBS/desulfurized rubber powder composite reduces material costs by 15% and significantly improves the moisture damage resistance of the asphalt mixture compared to SBS modification alone [24].

However, SBS-modified asphalt is prone to performance degradation and segregation under prolonged high-temperature construction [25,26]. In contrast, Styrene–Ethylene–Butylene–Styrene (SEBS) as a hydrogenated derivative of SBS exhibits higher chemical inertness due to the saturation of its double bonds. This saturated structure, devoid of unsaturation, grants SEBS greater rigidity and lower polarity [27]. Consequently, it demonstrates superior performance in high-temperature rutting resistance, aging resistance, and storage stability, though it exhibits comparatively poorer low-temperature performance.

Therefore, this study combined SEBS and SBS to form SEBS/SBS (SE-S) and investigated the effect of the SE-S system on asphalt performance for the first time. Then, the activated desulfurized rubber powder (ARP) was prepared via mechanical thermal oxidation and used to produce ARP/SE-S composite-modified asphalt (ASSA) combined with SE-S. Then, the conventional and rheological properties of ASSA with different RAP contents were tested and comprehensively compared with base asphalt (BA), ARP-modified asphalt (ARA), SBS-modified asphalt (SBSMA), and SE-S-modified asphalt (SE-SMA). Finally, the dispersion characteristics and modification mechanism of ASSA were investigated through microscopic morphology testing and infrared spectroscopy analysis.

## 2. Materials and Methods

### 2.1. Materials

#### 2.1.1. Base Asphalt

The 70# base asphalt (BA) from Sinopec Qilu Petrochemical Company in Zibo, China was used in this study. Its basic properties met the specification requirements, as shown in Table 1.

#### 2.1.2. Rubber Powder

**Figure 1:**
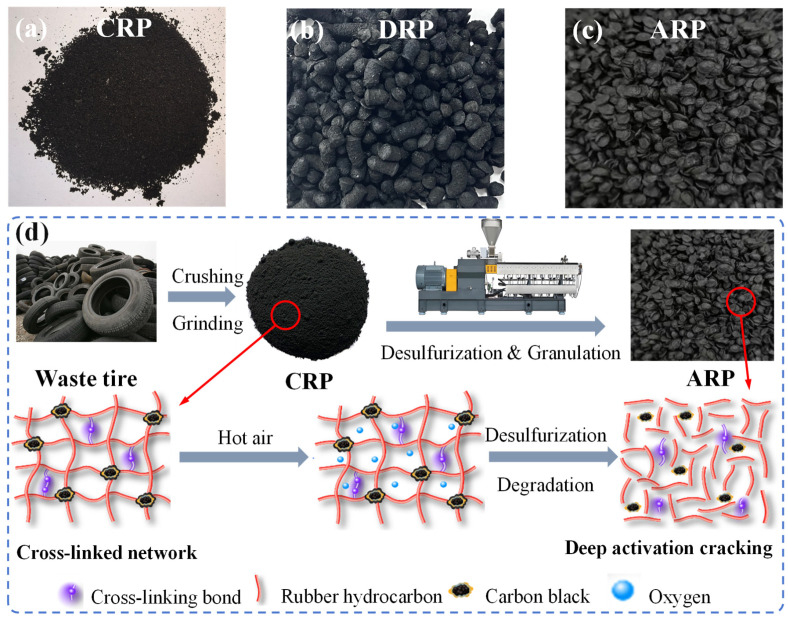
The appearance of the rubber powder and the production process diagram of ARP. (**a**) CRP; (**b**) DRP; (**c**) ARP; (**d**) The production process diagram of ARP.

The rubber powder used in this study includes crushed rubber powder (CRP) without desulfurization, traditional desulfurized rubber powder (DRP), and activated desulfurized rubber powder (ARP). The ARP used in the study was acquired through a mechano-thermal-oxygen non-additive desulfurization process. The basic indicators of ARP are listed in Table 2. The appearance of ARP is shown in Figure 1a–c.

The production process of ARP is outlined as follows: (1) Pre-treatment: Waste tires are crushed and screened through a grinder to produce crumb rubber powder (CRP). The CRP is then mixed with a specific proportion of softener at ambient temperature to facilitate subsequent mechanical desulfurization. (2) Core Desulfurization: The CRP is continuously fed into a twin-screw extruder. High-temperature hot air is injected via an air injection system, while the screw rotation speed is maintained between 80 and 100 r/min. Within the enclosed reaction environment, the crumb rubber undergoes stepwise activation and desulfurization. The temperature during the feeding stage is controlled at 80–100 °C, and at 180–210 °C during the compression stage. In the deep desulfurization stage, the temperature is raised to 200–250 °C. Under the combined action of residual oxygen and intense shear forces, the crosslinked network is further broken down and the material is homogenized, thereby completing the deep desulfurization and refining process. (3) Cooling and Pelletizing: The desulfurized rubber extruded from the extruder is immediately cooled (to 50–60 °C) to quench the oxidative reaction, prevent excessive degradation, and stabilize its plastic structure. Finally, the material is pelletized to obtain the ARP. The production process diagram of ARP is shown in Figure 1d.

#### 2.1.3. SBS and SEBS

**Figure 2:**
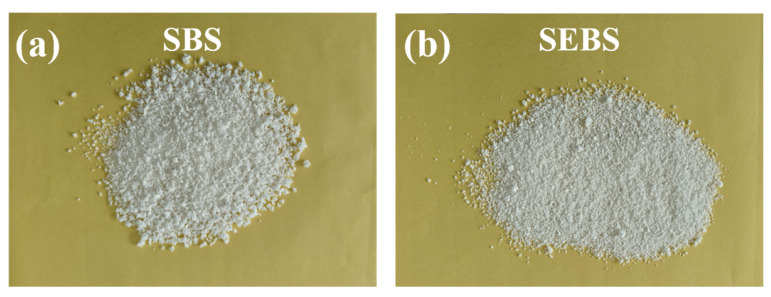
The appearance of SBS and SEBS. (**a**) SBS; (**b**) SEBS.

The SBS and SEBS used in the study were produced by Sinopec Hunan Petrochemical Co., Ltd. in Yueyang, China, with the models YH-791 and YH-503, respectively. Their appearance is shown in Figure 2, and the basic indicators are shown in Table 3.

#### 2.1.4. Additives

The additives used in this study include solubilizer and stabilizer. The solubilizer is liquid rubber oil, model Naphthenic acid 4010, sourced from Henan Leimo Chemical Products Co., Ltd. in Zhengzhou, China. It is used to improve the compatibility between the modifier and the asphalt. In this study, a low dosage of sulfur (0.2 wt.%) was used as a stabilizer for the modified asphalt. It is used to form crosslinked structures within SBS/SEBS polymer chains to enhance the stability of modified asphalt and reduce phase separation during storage.

#### 2.1.5. Preparation of Modified Asphalt

The modified asphalt used in this study includes SBSMA, SE-SMA, ARA, and ASSA with different ARP dosages. The dosage of ARP used in the ARA was 20%. A high-speed shearing machine and mixer were used to prepare modified asphalt in this study.

**Figure 3:**
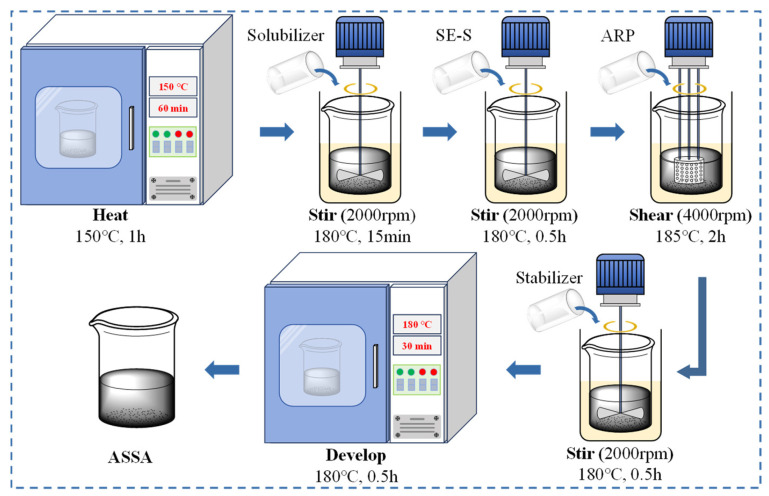
The preparation process of ASSA.

The ASSA was prepared according to the following procedure: (1) The base asphalt was heated in an oven at 150 °C for 1 h until it became fluid. (2) The solubilizer was blended into the base asphalt using a high-speed disperser at 180 °C and 2000 rpm for 15 min. (3) SE-S was added to the mixture, and blending was continued under the same conditions (180 °C, 2000 rpm) for another 30 min. (4) The mixture was subsequently sheared using a high-shear mixer at 185 °C and 4000 rpm for 2 h. (5) The stabilizer was incorporated into the asphalt blend using the high-speed disperser at 180 °C and 2000 rpm for 30 min. (6) Finally, the resulting modified asphalt was placed in an oven at 180 °C for 30 min to allow for swelling and development. The preparation process of ASSA is shown in Figure 3.

### 2.2. Test Methods

#### 2.2.1. Conventional Performance Test of Asphalt

According to the “Standard Test Methods of Bitumen and Bituminous Mixtures for Highway Engineering” (JTG E20-2011), the conventional performance of the asphalt in this study was tested, including ductility, penetration, softening point, and Brookfield viscosity [28]. The tests were repeated at least 3 times for each sample, and the average value was reported.

#### 2.2.2. Rheological Test of Asphalt

(1) Temperature scanning test

The temperature scanning mode of the dynamic shear rheometer (DSR) was used for temperature scanning tests of asphalt. According to the American Association of State Highway and Transportation Officials (AASHTO) standard, “Standard Method of Test for Determining the Rheological Properties of Asphalt Binder Using a Dynamic Shear Rheometer (DSR)” (AASHTO T 315-2024), temperature scanning tests were conducted on asphalt binders [29]. The test temperature started at 52 °C and increased to 88 °C in 6 °C intervals. The applied strain level was 12% and the testing frequency was 10 rad/s. The complex shear modulus (G*) and phase angle (δ) at each temperature were recorded during the tests. The rutting factor (G*/sin (δ)) of asphalt can be calculated through G* and δ. The temperature scanning tests were performed on a minimum of 3 freshly prepared specimens for each binder type to ensure reliability. The curves depict the mean response.

(2) Frequency scanning test

Frequency scanning tests were used to evaluate the viscoelastic rheological properties of asphalt. According to the AASHTO standard, “Standard Method of Test for Determining the Rheological Properties of Asphalt Binder Using a Dynamic Shear Rheometer (DSR)” (AASHTO T 315-2024), frequency scanning tests were conducted on asphalt binders [29]. The test temperature was 64 °C, which better matches the high temperature of an actual road surface. The applied strain level was 1%, and the frequency scanning range was 0.1–100 rad/s. The frequency scanning tests were performed on a minimum of 3 freshly prepared specimens for each binder type to ensure reliability. The curves depict the mean response.

(3) Multiple stress creep recovery (MSCR) test

The MSCR test was developed to provide a more accurate approach for evaluating the permanent deformation resistance of asphalt binder. According to the AASHTO standard, “Performance-Graded Asphalt Binder Using Multiple Stress Creep Recovery (MSCR) Test” (AASHTO M 332-2023), MSCR tests were conducted on asphalt binders [30]. The loading and unloading tests on asphalt were performed repetitively at different stress levels. The two stress levels were 0.1 kPa and 3.2 kPa, with 10 cycles per stress level. Each cycle included a 1 s loading process and a 9 s unloading recovery process for asphalt. The test temperature was 60 °C. The time-strain (γ − t) relationship of asphalt was obtained through MSCR tests. The MSCR tests were performed on a minimum of 3 freshly prepared specimens for each binder type to ensure reliability. The average value was reported.

(4) Bending beam rheometer (BBR) test

The BBR test was used to evaluate the low-temperature crack resistance performance of asphalt binder. According to the AASHTO standard, “Standard Method of Test for Determining the Flexural Creep Stiffness of Asphalt Binder Using the Bending Beam Rheometer (BBR)” (AASHTO T 313-2019), BBR tests were conducted on asphalt binders [31]. The test temperatures were −12 °C, −18 °C, and −24 °C. The creep stiffness modulus (S) and creep rate (m) of asphalt were determined through BBR tests. The low-temperature grades of the asphalt samples were determined under the conditions of S ≤ 300 MPa and m ≥ 0.3. The BBR tests were performed on a minimum of 3 freshly prepared specimens for each binder type to ensure reliability. The average value was reported.

#### 2.2.3. Microscopic Performance Test of Asphalt

(1) Scanning electron microscopy (SEM) test

SEM tests were used to observe the microstructure differences between modified asphalt samples. The dispersion and dissolution states of rubber powder in asphalt are closely related to their surface structures. Before observation, the samples were treated by spraying them with gold, and then the samples were observed at an accelerating voltage of 5 kV. The selected magnification for comparison testing was 1000×. The displayed images are representative results obtained from multiple observations of different regions of the sample.

(2) Fluorescence microscopy (FM) test

FM tests were used to observe the structure of asphalt and the distribution of the modifiers in asphalt. The compatibility between the modifiers and asphalt was evaluated based on their distribution patterns. The selected magnification for comparison testing was 40×. The displayed images are representative results obtained from multiple observations of different regions of the sample.

(3) Fourier transform infrared spectroscopy (FTIR) test

FTIR tests were used to measure the size, intensity, and position of the absorption peaks in asphalt. The reactions during the modification process were predicted by observing the changes in functional groups. The selected wavenumber range is 4000–600 cm^−1^, with a resolution of 4 cm^−1^. The sample was laid on the operating platform and scanned 64 times at a resolution of 4 cm^−1^. The FTIR tests were performed on a minimum of 3 freshly prepared specimens for each binder type to ensure reliability.

## 3. Results and Discussion

### 3.1. Analysis of the Impact of SE-S on Asphalt Performance

#### 3.1.1. Optimization of the SE-S Composite Ratio

**Figure 4:**
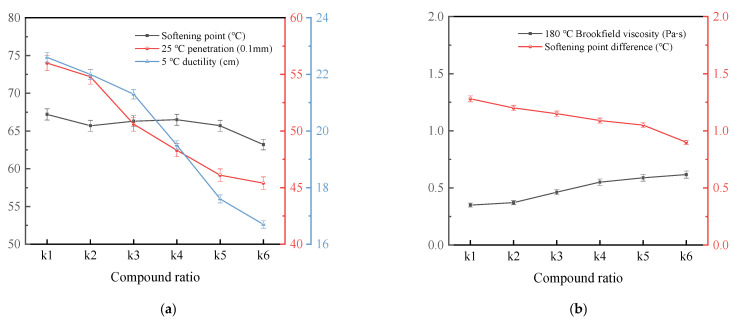
The effect of SE-S composite ratio on the performance of asphalt. (**a**) Softening point, 25 °C penetration and 5 °C ductility; (**b**) 180 °C Brookfield viscosity and softening point difference.

To determine the optimal SE-S composite ratio and investigate its effect on asphalt performance, SE-S-modified asphalts with a content of 4% were prepared and tested. The tested composite ratios of SEBS and SBS were k1 (0:1), k2 (0.2:0.8), k3 (0.4:0.6), k4 (0.6:0.4), k5 (0.8:0.2), and k6 (1:0). The results are presented in Figure 4.

As shown in Figure 4a, as the proportion of SEBS in SE-S increases, the 5 °C ductility of asphalt shows a decreasing trend. When the proportion of SEBS exceeds 40%, the magnitude of the decrease in ductility significantly increases. This indicates that an increase in the proportion of SEBS in SE-S will result in a decrease in the low-temperature performance of modified asphalt. In addition, SBS is superior to SEBS in improving low-temperature performance. The possible reason is that the hydrogenated structure of SEBS reduces the flexibility of polymer chains, thereby affecting their ability to deform at low temperatures [32]. The 25 °C penetration shows a similar variation pattern in elongation. The penetration decreases as the proportion of SEBS increases. Compared with the ratio of k1, the 25 °C penetration of modified asphalt decreased by about 17.7% at k5. This indicates that SBS can better maintain the flexibility of asphalt, while the addition of SEBS increases the overall stiffness of asphalt, manifested macroscopically as the hardening of asphalt. In addition, with the change in the composite ratio, the trend of the softening point of asphalt is gentle. This indicates that the ratio of SBS to SEBS has no significant effect on the high-temperature performance of modified asphalt.

Figure 4b reveals that increasing the SEBS proportion raises the 180 °C Brookfield viscosity, owing to the heightened difficulty in dispersing its saturated chains within asphalt, which increases molecular friction. Conversely, the concurrent decrease in the softening point difference underscores the superior role of SEBS in enhancing storage stability over SBS. The composite ratio of SEBS:SBS = 0.4:0.6 was therefore chosen for further study due to its balanced performance.

#### 3.1.2. Analysis of the Influence of SE-S Dosage on Asphalt Performance

**Figure 5:**
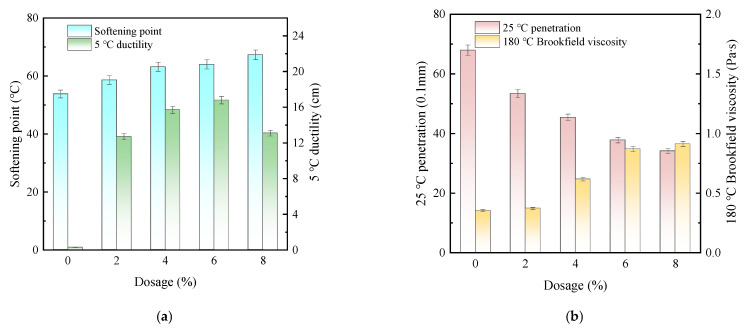
The effect of SE-S content on asphalt performance. (**a**) Softening point and 5 °C ductility; (**b**) 25 °C penetration and 180 °C Brookfield viscosity.

To evaluate the modification effect of SE-S, asphalt was modified with different SE-S dosages, and the corresponding performance results are shown in Figure 5.

The softening point, a key indicator of high-temperature deformation resistance of asphalt, consistently increases with SE-S content, as shown in Figure 5a. At 8% SE-S content, the softening point is approximately 25.1% higher than that of BA, demonstrating a significant improvement in high-temperature performance. This enhancement is attributed to the three-dimensional network formed by SBS and SEBS polymers within the asphalt. A higher SE-S dosage leads to a denser network, thereby more effectively restricting asphalt flow and improving high-temperature stability. As shown in Figure 5a, the base asphalt exhibits extremely low ductility and typical brittle behavior at low temperatures. The addition of 2% SE-S sharply increases the ductility to 12.7 cm, demonstrating that the polymer elastic network imparts excellent elastic deformation capability. However, when the SE-S dosage exceeds 6%, the ductility decreases. This phenomenon is likely due to insufficient polymer swelling in asphalt, which reduces the uniformity and flexibility of the crosslinked network [33].

As shown in Figure 5b, the penetration of the modified asphalt decreases significantly with increasing SE-S content, indicating the hardening of the asphalt system. This occurs as the polymer absorbs light oil components and its long-chain structure increases internal friction. Notably, the viscosity rises to 0.87 Pa·s at 6% SE-S, 2.5 times the base asphalt’s value, due to the entanglement and friction of polymer chains at high temperatures. While this enhanced viscosity improves the coating ability on aggregates, it also increases pumping resistance, mixing energy consumption, and demands stricter temperature control during construction. The increased internal resistance to flow may require a moderate increase in mixing and compaction temperatures to achieve sufficient coating of aggregates and proper densification of the asphalt mixture. This could lead to higher energy consumption and increased emissions of fumes during construction. The elevated viscosity, especially at standard pumping temperatures, would result in greater resistance during pumping, potentially demanding more powerful equipment. Considering both performance and economic factors, the modified asphalt demonstrates good comprehensive performance at an SE-S dosage between 2% and 4%.

### 3.2. Analysis of the Conventional Performance of ASSA

#### 3.2.1. Analysis of Basic Performance

**Figure 6:**
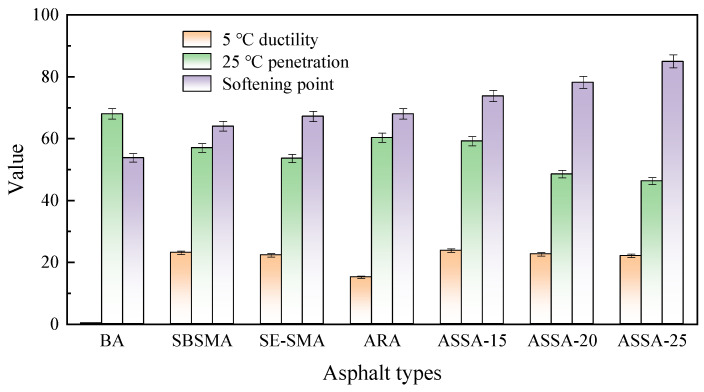
Test results of basic performance of asphalts.

To evaluate the synergistic effect of ARP and SE-S, the basic performance of ASSA with varying ARP dosages were tested and compared with those of BA, SBSMA, SE-SMA, and ARA, as shown in Figure 6.

As shown in Figure 6, all modified asphalts exhibit significantly lower penetration than BA. The 25 °C penetration of SE-SMA and ARA decreased by 21.2% and 11.3%, respectively, indicating enhanced cohesion and rutting resistance from ARP or SE-S. Furthermore, penetration decreases with higher ARP content, with ASSA-25 showing the lowest value which is 21.8% lower than ASSA-15. This is mainly owing to the ability of ARP to adsorb light asphalt components (aromatics and saturates) and form crosslinked structures, which enhances cohesion but also reduces lightweight components, resulting in asphalt hardening.

Compared to BA, all modified asphalts show significantly improved ductility, confirming that both SE-S and ARP form elastic networks that enhance deformation ability. While ARA exhibits lower ductility than SE-SMA and SBSMA, the addition of SE-S markedly improves this property. ASSA-15 shows a 56.6% increase over ARA, demonstrating significant synergy. This is because the SE-S/ARP combination creates a superior crosslinked structure, enhancing ductility. Moreover, increasing ARP content slightly reduces ASSA ductility but does not substantially impair low-temperature performance. Notably, ASSA exhibits a significantly higher softening point. For instance, the softening point of ASSA-15 is 37.2% and 9.8% higher than that of BA and SE-SMA, respectively, demonstrating the synergistic effect of ARP and SE-S in enhancing the high-temperature performance of asphalt.

#### 3.2.2. Analysis of Viscosity-Temperature Performance

**Figure 7:**
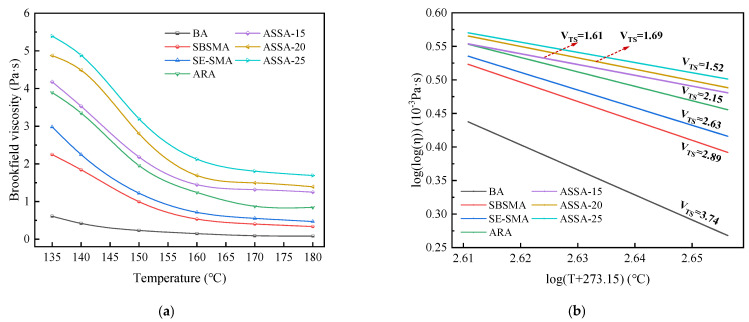
Test results of viscosity-temperature performance of asphalts. (**a**) Viscosity-temperature curves; (**b**) Fitting of viscosity-temperature curves.

To better characterize high-temperature performance, Brookfield viscosity was measured at various temperatures, as shown in Figure 7a. All modified asphalts showed higher viscosity than BA, following the order: ASSA > ARA > SE-SMA > SBSMA, confirming the thickening effect of ARP and SE-S. Although the viscosity of ASSA is notably higher at lower temperatures, the difference to SE-SMA and SBSMA becomes minimal above 170 °C, demonstrating improved workability. Furthermore, while viscosity decreases with temperature for all samples, ASSA exhibits a more pronounced decline between 135 and 150 °C, facilitating practical application.

The Refutas equation (Equation (1)) was fitted to the viscosity-temperature curves to quantify the temperature susceptibility of the binders via the V_TS_ parameter, as shown in Figure 7b. BA shows the highest V_TS_, indicating the greatest temperature sensitivity. In contrast, the V_TS_ values of SBSMA and SE-SMA are 22.7% and 29.7% lower than that of BA, respectively, demonstrating the effectiveness of polymer modification in reducing thermal susceptibility. Furthermore, ASSA exhibits even lower V_TS_, which decreases further with increasing ARP content, confirming the significant synergistic effect of SE-S and ARP in enhancing the temperature stability of asphalt.

(1) $$\mathrm{lg}[\mathrm{lg}(\eta \times 10^{3})]=n-V_{TS}\mathrm{lg}(T)$$

where η is the Brookfield viscosity, Pa·s; T is the experimental temperature, K; n and V_TS_ are the parameters to be fitted.

#### 3.2.3. Analysis of Storage Stability

**Figure 8:**
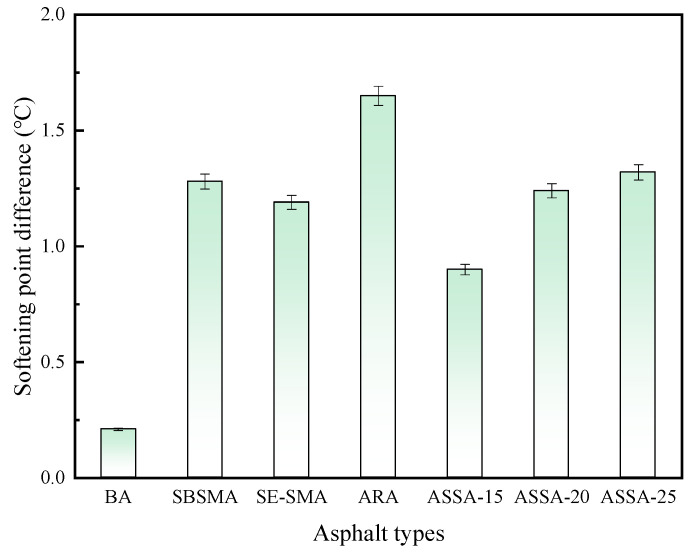
Test results of storage stability of asphalts.

The storage stability of the modified asphalt, which is critical for performance uniformity and pavement service life, was assessed by the softening point difference test. The results are presented in Figure 8.

As shown in Figure 8, ARA exhibits the highest softening point difference value, indicating poor storage stability. This is due to an insufficient shearing process resulting in the presence of small particles of rubber powder. Driven by thermodynamic incompatibility, a phase separation process dominated by the physical settling of rubber powder particles occurred due to significant density differences and a decrease in asphalt viscosity at high temperatures. The softening point difference values of SBSMA and SE-SMA are smaller, indicating that the addition of SEBS and SBS can effectively improve the storage stability of asphalt. The addition of SE-S increases the viscosity of modified asphalt, hinders particle movement and settling, and reduces segregation. It is worth noting that ASSA exhibits better storage stability compared to ARA. The softening point difference value of ASSA-20 has decreased by 24.8% compared to ARA. This is because the synergistic effect of ARP and SE-S forms a stable crosslinked structure in asphalt, which limits the settling and movement of rubber powder particles in asphalt. In addition, with the increase in ARP dosage, the movement of rubber powder particles intensifies, leading to a decreasing trend in the storage stability of ASSA.

### 3.3. Analysis of Rheological Properties of ASSA

#### 3.3.1. Analysis of Temperature Scanning Test

**Figure 9:**
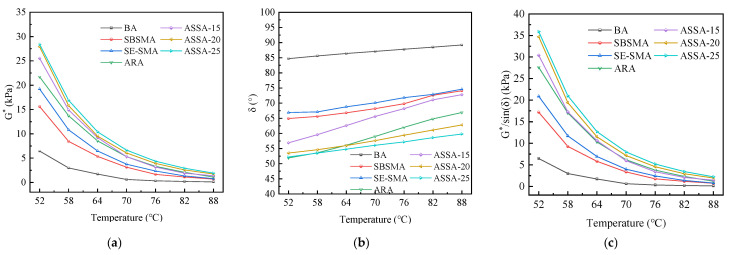
Temperature scanning test results of asphalts. (**a**) G*; (**b**) δ; (**c**) G*/sin (δ).

Temperature scanning tests were performed on various asphalt types to evaluate their viscoelastic behavior. The resulting curves, which depict the relationships between complex shear modulus (G*), phase angle (δ), and the rutting factor (G*/sinδ) as functions of temperature, are presented in Figure 9.

As illustrated in Figure 9a, G* exhibits a clear decreasing trend with rising temperature. The base asphalt demonstrates the lowest G* value, indicating that the addition of modifiers enhances the asphalt’s stiffness and deformation resistance under load. At the same temperature, the G* of ASSA is significantly higher than that of SE-SMA and SBSMA. Furthermore, the G* of ASSA increases with a higher dosage of ARP. This evidence suggests that the synergistic interaction between ARP and SE-S fosters the formation of a stable crosslinked structure within the asphalt, thereby improving its viscoelastic properties and overall deformation resistance [34].

As shown in Figure 9b, the phase angle of asphalt increases with rising temperature. Compared to base asphalt (BA), the modified asphalts exhibit lower δ values, suggesting that the modifiers enhance the elastic proportion of the material. The higher δ of SE-SMA compared to SBSMA and ASSA is attributed to the significant energy dissipation from internal friction within its heterogeneous microstructure comprising SEBS and SBS phases. This friction, stemming from the interaction between incompatible phases, increases the viscous component of the response, thereby elevating the phase angle. In contrast, ASSA shows a lower δ than SE-SMA, indicating that the incorporation of ARP further enhances the elastic behavior of the modified asphalt. This effect becomes more pronounced with increasing ARP content, leading to an improved elastic ratio. While ARA shows comparable or lower phase angles than ASSA-20/25 at low temperatures (50–60 °C), it surpasses them as the temperature increases. The crossover in phase angle underscores a transition from a low-temperature regime dominated by the homogeneous, rigid ARP network in ARA to a high-temperature regime where the stable SBS/SEBS network in the ASSA blends ensures superior elastic retention.

Figure 9c shows that G*/sinδ follows a trend similar to G*, decreasing with temperature. This decline is attributed to the weakening of molecular crosslinking and the reduction in the elastic component as temperature rises. Notably, ASSA demonstrates the highest G*/sinδ values among the tested materials at the same temperature. This confirms that the combination of ARP and SE-S robustly improves rutting resistance, with the enhancement being directly proportional to the ARP dosage.

#### 3.3.2. Analysis of Frequency Scanning Test

**Figure 10:**
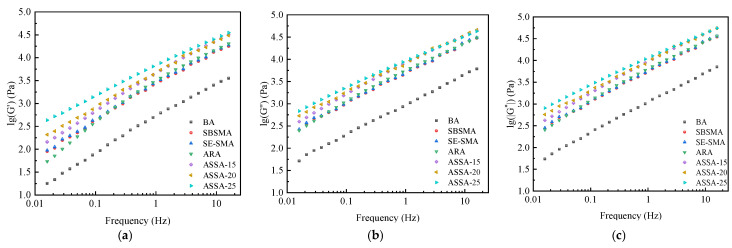
Frequency scanning test results of asphalts. (**a**) Storage modulus; (**b**) Loss modulus; (**c**) Main curve of complex modulus.

Frequency scanning tests on various asphalts were conducted to simulate pavement responses at different driving speeds and to evaluate high-temperature performance. The results are shown in Figure 10.

As shown in Figure 10a, G′ increases with loading frequency across all samples, with ASSA exhibiting the highest values, further boosted by a greater ARP content. This confirms that the ARP and SE-S combination markedly improves elasticity and deformation recovery. Figure 10b shows a parallel increase in G″ with frequency, attributed to intensified internal friction. Notably, the loss modulus curve for ASSA becomes progressively smoother with higher ARP dosage, indicating that ARP effectively mitigates the asphalt’s sensitivity to variations in loading frequency.

Figure 10c displays the main curves of the complex shear modulus for all asphalt. The main curves exhibit a similar upward trend with increasing reduced frequency, with the differences between them gradually diminishing. At higher frequencies, the curves converge, approaching the glassy modulus of asphalt. ASSA exhibits a significantly higher modulus than SBSMA and SE-SMA, particularly in the low-frequency range. This behavior suggests that SBS and SE-S asphalts are more sensitive to frequency and temperature changes. Given that pavement service primarily involves mid-to-low frequency loading, the enhanced modulus of ASSA in this range is practically advantageous. Consequently, the frequency scanning analysis validates that ASSA offers the best high-temperature performance, corroborating the temperature scanning findings and confirming the efficacy of the ARP and SE-S combination.

#### 3.3.3. Analysis of High-Temperature Resistance to Permanent Deformation Performance

**Figure 11:**
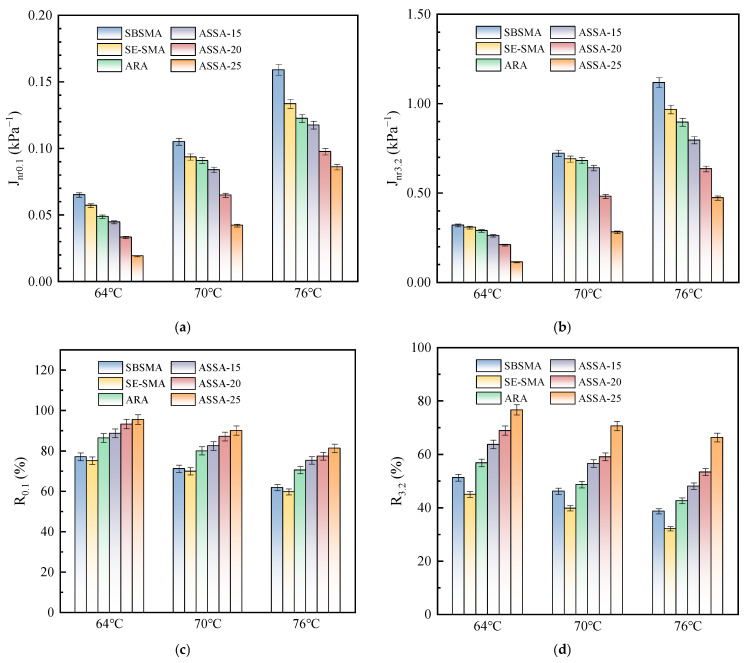
MSCR test results of asphalts. (**a**) J_nr0.1_; (**b**) J_nr3.2_; (**c**) R_0.1_; (**d**) R_3.2_.

To evaluate the high-temperature resistance to permanent deformation of ASSA, MSCR tests were conducted to analyze the creep and recovery behavior of modified asphalt under different stress levels. The tests were performed at 64 °C, 70 °C, and 76 °C under low (0.1 kPa) and high (3.2 kPa) stress conditions. The creep recovery rate (R_0.1_ and R_3.2_) and non-recoverable creep compliance (J_nr0.1_ and J_nr3.2_) of the modified asphalt were calculated from the test data. The results are presented in Figure 11.

The J_nr_ is a critical metric for evaluating resistance to permanent deformation, where a lower value indicates better performance. As shown in Figure 11a,b, the variation of J_nr_ demonstrates a consistent trend across different loading conditions. Specifically, ARA and ASSA exhibit significantly lower J_nr_ values compared to SBSMA and SE-SMA. For instance, at 64 °C, the J_nr0.1_ values of ARA and ASSA-15 are 25.1% and 31.5% lower than that of SBSMA, respectively, confirming that ARP addition substantially enhances deformation resistance. Furthermore, the J_nr_ value of ASSA decreases with increasing ARP content. This improvement is attributed to the ARP, which swells by absorbing light components in the asphalt and, under the action of a crosslinking agent, reconstructs a robust polymer network to resist deformation. Concurrently, the synergistic effect between SE-S and ARP stabilizes this crosslinked structure, further augmenting the high-temperature rheological properties of the asphalt [26].

The R quantifies the elastic recovery of asphalt, with higher values denoting a larger elastic component and better performance. Figure 11c,d show that R decreases with temperature for all samples due to reduced elastic recovery at high temperatures. Conversely, the R value of ASSA increases significantly with ARP content, especially under 3.2 kPa stress. At 70 °C, the R_3.2_ value of ASSA-20 is 48.5% and 21.3% higher than that of SE-SMA and ARA, respectively. This demonstrates that ARP enhances elasticity, while SE-S mitigates the viscous flow of ARA, collectively granting ASSA superior recovery and load-bearing capacity.

#### 3.3.4. Analysis of Low-Temperature Crack Resistance Performance

**Figure 12:**
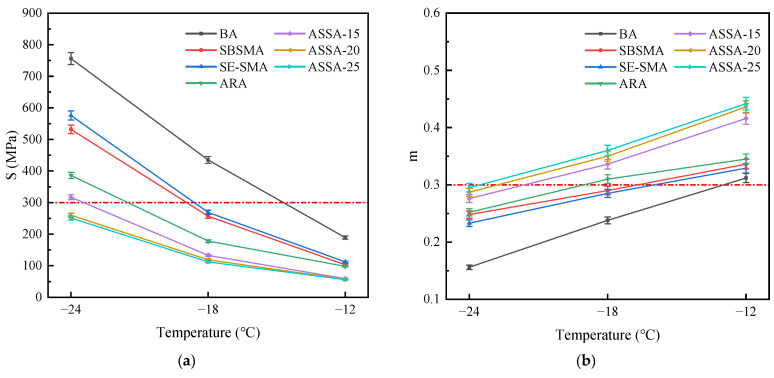
BBR test results of asphalts. (**a**) S; (**b**) m.

To evaluate the low-temperature crack resistance of the modified asphalts, BBR tests were conducted to measure their rheological properties and stress relaxation under a fixed load. The resulting stiffness modulus (S) and creep rate (m) for each asphalt are shown in Figure 12.

Asphalt is prone to low-temperature cracking in service due to reduced viscoelasticity. The Strategic Highway Research Program (SHRP) specifications stipulate that asphalt exhibits good low-temperature crack resistance when its S value is ≤300 MPa and its m value is ≥0.3. Figure 12a shows that modified asphalts have markedly lower S values than BA. The S value drops exponentially as temperature rises. While all asphalt samples remain flexible at −12 °C, a pronounced difference in S values between ASSA and other binders emerges as the temperature drops to −24 °C. This indicates the superior performance of ASSA, particularly under more severe low-temperature conditions. At −24 °C, only ASSA-15 and ASSA-20 meet the criterion with S values below 300 MPa. Moreover, ASSA consistently shows lower S values than ARA and SE-SMA at the same temperature. For example, the S value of ASSA-15 is 25.3% lower than that of ARA and 50.6% lower than that of SE-SMA at −18 °C. This confirms that the ARP/SBS synergy more effectively boosts deformation resistance than either modifier alone. It is also notable that a higher ARP content leads to a lower S value in ASSA. This trend is likely due to improved elasticity and ductility from the rubber, which enhances low-temperature performance. Furthermore, increased rubber powder promotes a networked crosslinking structure in the asphalt, significantly improving its resistance to low-temperature cracking.

The m value quantifies the stress relaxation ability of asphalt at low temperatures, with a higher value indicating more viscous components and thus better flexibility and deformation resistance. Figure 12b shows that at identical temperatures, the m values rank as follows: ASSA > ARA > SBSMA > SE-SMA. The S-value of SE-SMA is lower than that of SBSMA, indicating poorer low-temperature performance. This is due to the hydrogenated structure of SEBS, which stiffens the polymer chains and limits their low-temperature deformation. The m value decreases with temperature. While most asphalts have an m value above 0.3 at −12 °C, only ARA and ASSA retain this performance at −18 °C, highlighting the superior stress relaxation capability of ASSA in colder conditions. ARP and SE-S create a denser crosslinked network, which reduces molecular slip, increases the creep rate, and improves low-temperature performance. This enhancing effect is more significant with higher ARP dosage. It is worth noting that the low-temperature performance of ASSA-25 is not significantly improved compared to ASSA-15 and ASSA-20. The very high ARP content absorbs a large number of light components from the asphalt, which can lead to a slight hardening of the overall matrix, counteracting the benefits of increased elasticity.

### 3.4. Analysis of Microstructure and Modification Mechanism

#### 3.4.1. Analysis of Fluorescence Dispersibility

**Figure 13:**
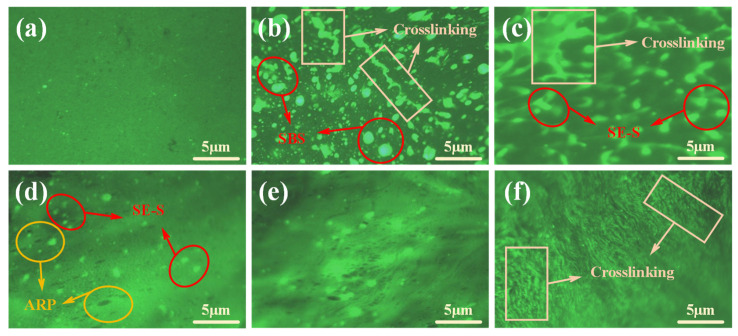
Fluorescence microscopy test result of asphalts. (**a**) BA; (**b**) SBSMA; (**c**) SE-SMA; (**d**) ASSA-15; (**e**) ASSA-20; (**f**) ASAA-25.

To evaluate the dispersibility and compatibility of the modifier in the asphalt, the microstructure of the modified asphalt was examined using fluorescence microscopy, as shown in Figure 13.

As shown in Figure 13, the modifiers exhibit characteristic fluorescence responses under UV excitation. SBS and SEBS appear as white luminescent spots, while ARP is distinguished by black spots. The base asphalt presents a dark green background due to the fluorescence quenching effect, creating a significant optical contrast. As seen in Figure 13b,c, both SBS and SEBS are uniformly dispersed as fine particles within the asphalt following high-speed shearing. Concurrently, these modifiers swell in the asphalt and progressively form a crosslinked network structure, establishing a distinct continuous phase. It is noteworthy that the crosslinked structure in SE-SMA is denser than that in SBSMA. This enhanced structural density is a key factor contributing to the improved high-temperature performance and storage stability of the modified asphalt.

As shown in Figure 13d, both SE-S and ARP in ASSA-15 are distinctly visible as granular particles and are evenly distributed throughout the asphalt. As the ARP dosage increases, the number of discrete particles in ASSA diminishes, and the boundaries between the modifiers become less distinct. At an ARP content of 25%, the modifiers merge to form a significant continuous phase within the asphalt. This morphological evolution indicates that SE-S and ARP undergo sufficient swelling and crosslinking within the asphalt. The excellent compatibility between the modifiers and the base asphalt contributes to a more stable ASSA system, resulting in its outstanding storage stability.

#### 3.4.2. Analysis of Microscopic Morphology

**Figure 14:**
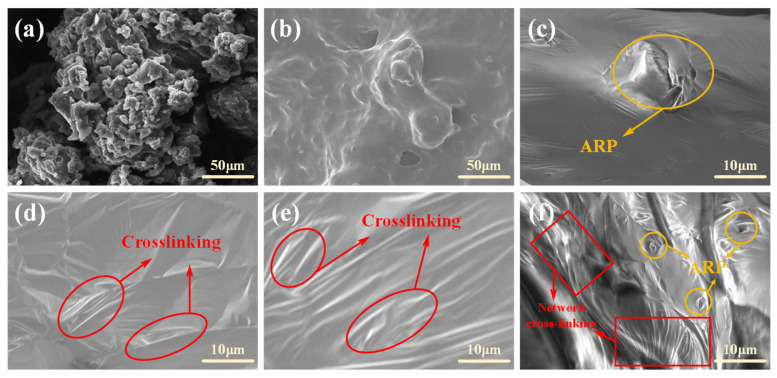
Test result of SEM. (**a**) CRP; (**b**) ARP; (**c**) ARA; (**d**) SBSMA; (**e**) SE-SMA; (**f**) ASAA.

The microstructure of the rubber powder and modified asphalt was characterized using SEM to observe the morphologies of the modifiers and the multiphase dispersion of polymers within the asphalt. The results are presented in Figure 14.

The SEM images in Figure 14a,b compare the microstructures of CRP and ARP. Both materials exhibit clustered distributions, but CRP particles appear more discrete with distinct angular features, attributable to mechanical shear crushing which preserves the high hardness and elasticity of waste tires. This morphology contributes to the poorer solubility of CRP in asphalt, leading to issues such as internal friction, agglomeration, high viscosity, and eventual segregation in CRP-modified asphalt. In contrast, ARP displays a fluffy, flocculent aggregate form with a smoother surface and lower particle dispersion. Desulfurization breaks its crosslinking bonds (e.g., C-S, S-S), creating a more adhesive surface [35]. Consequently, ARP demonstrates superior compatibility with asphalt, facilitating the formation of stable crosslinked structures, enhancing interfacial strength, and ultimately improving asphalt performance. This fundamental difference is key to the low segregation observed in ASSA.

Figure 14c displays the microstructure of ARA, where the surface shows discernible protrusions corresponding to partially swollen rubber particles, set against a comparatively smooth background. This morphology indicates favorable compatibility between ARP and the asphalt. As shown in Figure 14d,e, both SBSMA and SE-SMA exhibit similar surface topographies. Their surfaces are relatively smooth but feature distinct, angular wrinkles that are interconnected in a striated pattern. This wrinkled structure is attributed to the crosslinked network formed by SBS or SE-S within the base asphalt, which acts to restrain the flow of the asphalt and enhance its stability. As shown in Figure 14f, ASSA displays a more prominent and networked wrinkled structure compared to SBSMA and SE-SMA, alongside a minor presence of undissolved ARP. This indicates that most ARP swells and synergizes with SE-S to form a dense, crosslinked network that restricts asphalt flow, enhancing stability. This structure significantly boosts high-temperature deformation resistance and elastic recovery. The remaining ARP particles act as “elastic islands,” impeding micro-crack propagation to improve fatigue and low-temperature performance while reinforcing the mechanical strength and durability of the system.

#### 3.4.3. Analysis of Modification Mechanism

**Figure 15:**
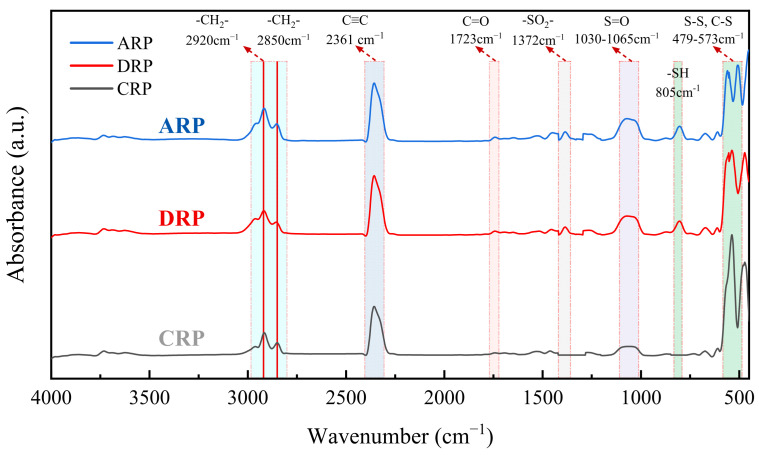
FTIR test results of rubber powder.

The molecular structures of ARP, DRP, and CRP were analyzed by FTIR to probe structural changes induced by the activation and desulfurization process. The results are shown in Figure 15.

As shown in Figure 15, compared with CRP, both ARP and DRP exhibit new absorption peaks at 1372 cm^−1^ and 805 cm^−1^, which are characteristic of thioesters and sulfhydryl (-SH) groups, respectively. These peaks are recognized in related studies as indicative of cleaved sulfur crosslinking bonds. Furthermore, the characteristic peaks for carbonyl (C=O) at 1723 cm^−1^ and sulfoxide (S=O) in the 1030–1065 cm^−1^ range are more pronounced in ARP and DRP than in CRP, whereas the absorption peaks for disulfide (S-S) and C-S bonds in the 479–573 cm^−1^ region are attenuated. These spectral changes suggest that during the desulfurization process, C-C, C-S, and S-S bonds are disrupted and broken, generating free radicals that subsequently undergo oxidative degradation with hot air. This confirms that the activation–desulfurization process breaks some sulfur-containing crosslinks, producing sulfur radicals that combine with atmospheric oxygen under the combined action of heat and shear forces, ultimately forming sulfonic acid or sulfone compounds. It is noteworthy that the typical absorption peaks of natural rubber at 2920 cm^−1^ and 2850 cm^−1^ remain highly consistent across all three rubber powders. This indicates that the desulfurized rubber largely retains the fundamental composition of natural rubber, and its core functional groups have not been severely compromised [36].

**Figure 16:**
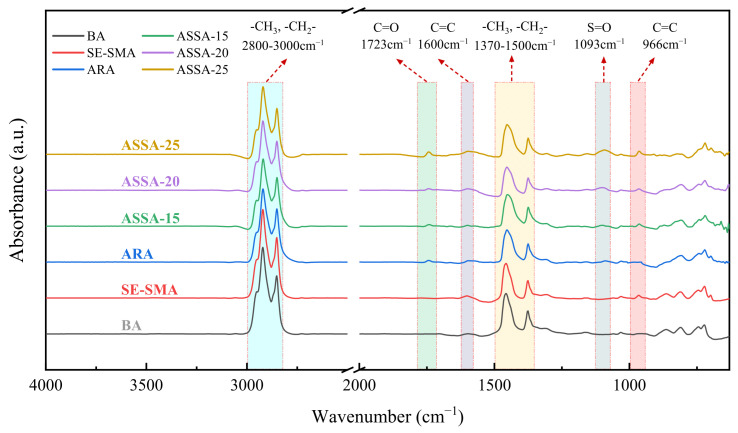
FTIR test results of asphalts.

Figure 16 shows the infrared spectra of different types of asphalt. The modified asphalts exhibit no significant differences in characteristic peaks compared to the BA in the regions of 2800–3000 cm^−1^ (-CH_n_), 1370–1500 cm^−1^ (-CH_n_), and 1600 cm^−1^ (C=C). However, ARA and ASSA display stronger absorption peaks at 1723 cm^−1^ (C=O) and 1093 cm^−1^ (S=O). The intensity of these peaks increases with a higher ARP content in ASSA, which is attributed to the reaction between the rubber powder and oxygen in hot air after the cleavage of crosslinking bonds during the activation and desulfurization process. This observation is consistent with the FTIR analysis of the rubber powder. Notably, distinct absorption peaks at 966 cm^−1^ are observed in SE-SMA and ASSA but are absent in BA and ARA. This peak, identified as the out-of-plane bending vibration of C=C, is characteristic of SBS and SEBS. A comparison with Figure 15 indicates that the main characteristic peaks of ASSA remain largely unchanged from those of the base asphalt. The modification mechanism of ASSA is predominantly physical, involving the formation of a crosslinked network through physical swelling and entanglement. The chemical changes observed by FTIR are attributed to the activation and desulfurization of the rubber powder, which enhances its compatibility and facilitates the physical modification process.

## 4. Conclusions

This study synthesized ARP using a mechano-thermal oxidation method and subsequently fabricated a composite-modified asphalt (ASSA) with ARP, SEBS, and SBS. A comprehensive evaluation of its overall performance and modification mechanism was conducted through conventional performance tests, rheological characterization, and microstructural analysis, exploring its potential application in high-grade highways. The main conclusions are as follows:

(1) SBS demonstrates superior efficacy in enhancing the low-temperature performance of asphalt, whereas SEBS more effectively improves the storage stability of the modified asphalt. With SEBS:SBS = 0.4:0.6 and a SE-S content of 2–4%, the modified asphalt exhibited better overall performance.
(2) The synergistic effect of ARP and the SE-S elastic medium significantly improves the creep recovery ability and low-temperature crack resistance of modified asphalt, resulting in the excellent viscoelastic rheological properties of ASSA.
(3) ARP increases the proportion of elasticity and ductility of asphalt. The addition of SE-S compensates for the high-viscosity deformation of ARA at higher temperatures, giving ASSA better low-temperature crack resistance and high-temperature deformation resistance.
(4) SE-S and ARP fully swelled and crosslinked in the asphalt, exhibiting excellent compatibility and endowing the ASSA with superior stability and performance.
(5) The modification mechanism of ASSA is predominantly physical, involving the formation of a crosslinked network through physical swelling and entanglement. The chemical changes observed by FTIR are attributed to the activation and desulfurization of the rubber powder, which enhances its compatibility and facilitates the physical modification process.

This study establishes that the strategic use of a SEBS/SBS composite system (SE-S) presents a viable and enhanced strategy for utilizing waste rubber. This approach not only contributes to environmental sustainability but also decisively improves the comprehensive performance of modified asphalt, particularly in balancing storage stability with high- and low-temperature properties. The ASSA demonstrates superior storage stability and high- and low-temperature performance compared to conventional SBSMA, making it suitable for high-grade highway applications. Although the composite modifier may incur a slightly higher initial cost, the use of waste tires enhances environmental sustainability, while improved durability may reduce long-term maintenance costs, indicating promising application potential. Field trials and life-cycle cost analysis will be conducted for further validation. The research findings are currently being translated into practical engineering applications. Future work will focus on increasing the rubber powder content to further improve the efficiency of waste recycling and explore its fatigue damage characteristics and long-term aging behavior.

## Figures and Tables

**Table 1:** Basic indicators of 70# BA.

Project		Requirements	Test Result
Penetration (25 °C, 100 g, 5 s) (0.1 mm)		60~80	68.7
Softening point (°C)		≥46	50.0
Ductility (5 cm/min, 10 °C) (cm)		≥20	80.7
Brookfield viscosity (135 °C) (Pa·s)		≥0.16	0.56
Dynamic viscosity (60 °C) (Pa·s)		≥160	248.3
After TFOT	Mass loss (%)	≤±0.8	0.49
	Penetration ratio (25 °C) (%)	≥61	65.7
	Ductility (5 cm/min, 10 °C) (cm)	≥8	10.3

**Table 2:** Basic indicators of ARP.

Project	Test Result
Relative density	1.18
Ash content (%)	7.2
Acetone extract (%)	11
Carbon black (%)	28.3
Rubber hydrocarbon (%)	51

**Table 3:** Basic indicators of SBS and SEBS.

Items	Value	
	SBS	SEBS
Model	YH-791	YH-503
Stress at 300% (MPa)	≥2	≥3
Elongation (%)	≥700	≥400
Permanent deformation (%)	≤40	≤40
Tensile strength (MPa)	≥15	≥16
Shore hardness (A)	≥68	≥70
Volatile component (%)	≤0.7	≤1.0

## Data Availability

The original contributions presented in this study are included in the article. Further inquiries can be directed to the corresponding author.

## Abbreviations

The following abbreviations are used in this manuscript:

SBS	Styrene–Butadiene–Styrene
SEBS	Styrene–Ethylene–Butylene–Styrene
CRP	crushed rubber powder
DRP	desulfurized rubber powder
ARP	activated desulfurized rubber powder
RA	rubber powder-modified asphalt
BA	base asphalt
SBSMA	SBS-modified asphalt
SE-S	SEBS/SBS
SE-SMA	SE-S-modified asphalt
ARA	ARP-modified asphalt
ASSA	ARP/SBS/SEBS composite-modified asphalt
ASSA-15	ASSA with an ARP content of 15%
ASSA-20	ASSA with an ARP content of 20%
ASSA-25	ASSA with an ARP content of 30%
